# Seasonal Metabolic Adaptations and Antioxidant Defense Mechanisms in the Resilience of *Rhynchosia minima* and *Senna italica* Legumes in Arid Region

**DOI:** 10.1155/sci5/1359373

**Published:** 2025-09-10

**Authors:** Rania Hamdy, Attiat Elnaggar, Najma Nur Islam, Sarah Sabri, François Mitterand Tsombou, Sameh S. M. Soliman, Ahmed M. Almehdi, Fouad Lamgharie, Kareem A. Mosa, Ali El-Keblawy

**Affiliations:** ^1^Research Institute for Science and Engineering (RISE), University of Sharjah, Sharjah 27272, UAE; ^2^Department of Botany and Microbiology, Faculty of Science, Alexandria University, Alexandria, Egypt; ^3^Department of Applied Biology, College of Sciences, University of Sharjah, P.O. Box 27272, Sharjah, UAE; ^4^Civil and Environmental Engineering, College of Engineering, University of Sharjah, Sharjah, UAE; ^5^Fujairah Research Centre (FRC), Al-Hilal Tower, P.O. Box 666, Fujairah 3003, UAE; ^6^Research Institute for Medical and Health Sciences (RIMHS), University of Sharjah, P.O. Box 27272, Sharjah, UAE; ^7^Department of Medicinal Chemistry, College of Pharmacy, University of Sharjah, P.O. Box 27272, Sharjah, UAE; ^8^College of Sciences, Department of Chemistry, University of Sharjah, P.O. Box 27272, Sharjah, UAE; ^9^Faculty of Pharmacy, Al Salam University, Tanta, Egypt

**Keywords:** antioxidant activity, metabolic profiling, *Rhynchosia minima*, *Senna italica*, stress adaptation

## Abstract

Plants survive the extreme seasonal and environmental conditions by developing various bioactive compounds. These compounds support their survival in harsh environments. This study examines how seasonal shifts influence the metabolic profiles and antioxidant responses of *Rhynchosia minima* and *Senna italica*, shedding light on their metabolic adaptation strategies to arid environments. Both species exhibited significant seasonal variations in total phenolic content (TPC), total flavonoid content (TFC), and antioxidant activity. Notably, *R. minima* displayed a 4.8% (0.05-fold) increase in TPC and a more pronounced 1.5-fold increase in TFC during winter compared to summer, while *S. italica* showed a 2.5-fold increase in TFC. Antioxidant activity was significantly higher in winter; *R. minima* exhibited a reduction from 98.34 μg/mL (summer) to 68.47 μg/mL (winter), and *S. italica* showed a decrease from 144.89 μg/mL (summer) to 84.25 μg/mL (winter), indicating enhanced bioactivity under cold stress. Although both species activate common cold stress metabolic pathways involving amino acids, lipids, and carbohydrates, they exhibit unique metabolic seasonal and species-specific patterns. Unique winter phenolic compounds from *R. minima* include epigallocatechin and 6-hydroxyflavone-β-*D*-glucoside, while cis-resveratrol and quercetin were unique to *S. italica*. *R. minima* demonstrates broader metabolic adaptations, with enriched metabolic pathways, such as glutathione metabolism, whereas *S. italica* relies on ubiquinone and α-linolenic acid metabolism. These findings provide insights into the intricate links between environmental stress, phytochemical adaptation, and ecological resilience of legume survival in the arid region, with a direction to antioxidant use in human health.

## 1. Introduction

Medicinal plants are indispensable sources of phytochemicals [1, 2], which are secondary metabolites that exert protective effects on plants and humans. It helps plants defend against pathogen and environmental stress. In human, they possess significant antioxidant, anti-inflammatory, and anticancer activity [3, 4]. Among these phytochemicals, phenolics are the most abundant and are considered key contributors to antioxidant activity due to their ability to neutralize reactive oxygen species (ROS), such as superoxide and hydroxyl radicals [5]. This antioxidant defense not only benefits plants but also offers protection against oxidative stress-related diseases in humans, including cardiovascular disorders, certain cancers, and age-related cellular damage [6].

*Rhynchosia minima* (*R. minima*) and *Senna italica (S. italica)* are members of the Fabaceae family, widely distributed in arid and semiarid regions of Africa and Asia. *Rhynchosia minima (R. minima)* and *Senna italica (S. italica)*, commonly known as Turvel, are twining or trailing annual plants belonging to the family Fabaceae, subfamily Papilionoideae [7]. They are widely distributed across tropical and subtropical regions, particularly in the arid and semiarid zones of Africa and Asia. Traditionally, its leaves have been used as an abortifacient in folk medicine and are known for their anthelmintic activity [8]. Other therapeutic applications include treatments for cuts, asthma, piles, and skin ailments [9]. Additionally, *S. italica*, a small shrub or herbaceous plant, has been used in traditional medicine for purgative properties and as a remedy for stomach aches, jaundice, fevers, venereal diseases, and intestinal disorders [10]. Its leaves are also used to treat skin conditions like ulcers and burns, while its roots are applied for colic, wound healing, and immunity-enhancing antioxidant and infantile diarrhea [10]. Their ecological resilience and medicinal relevance make them ideal candidates for investigating seasonal metabolic adaptations [10, 11].

Seasonal environmental changes including fluctuations in temperature, light, and water availability can impose stress on plants, often triggering increased biosynthesis of secondary metabolites like antioxidants to maintain cellular redox balance [12, 13]. These metabolic shifts are part of adaptive strategies that enable plants to cope with environmental stimuli, thereby contributing to the maintenance of cellular redox hemostasis and overall physiological function during periods of stress [14].

It is known that plants can alter their metabolite levels in response to environmental factors. However, this specific pattern of seasonal metabolic change in *R. minima* and *S. italica* legumes remains poorly studied. Previous studies have identified seasonal fluctuations in the levels of phenolic and flavonoid compounds during plant growth cycles [14], indicating that timing and environmental conditions play critical roles in determining phytochemical abundance. However, comprehensive data on how seasonal variation affects both the metabolic profile and antioxidant capacity in *R. minima* and *S. italica* specifically are lacking.

Given the practical and ecological importance of understanding such spatiotemporal dynamics, this study aims to systematically profile seasonal metabolic shifts in *R. minima* and *S. italica* two legumes well-adapted to arid environments to determine how these shifts align with antioxidant activity and environmental stress responses [15]. Therefore, this study aims to fill this knowledge gap by profiling seasonal metabolic changes in these two species and linking them to antioxidant activity and environmental stress across different seasons. Based on the premise that stress-related metabolites confer adaptive advantages, we hypothesized that the studied plants would accumulate higher levels of antioxidant-rich compounds during seasons with more extreme environmental conditions as a stress adaptation strategy [16]. To verify our hypothesis, we applied comparative metabolic analysis via gas chromatography–mass spectrometry (GC–MS), multivariate statistical analyses, and antioxidant activity assays across two contrasting seasonal conditions (summer and winter) for the two species.

We anticipate distinct metabolic adaptations in response to seasonable stress such as increased phenolic content and stronger antioxidant activity during the most challenging season. Such a finding would support the concept of seasonally regulated antioxidant response. Our work differs from existing research by offering the first comprehensive seasonal metabolomic profile of these two arid-region legumes, linking environmental stress with biochemical adaptation. The findings provide valuable insights for optimizing harvest strategies and maximizing the therapeutic potential of medicinal plants.

## 2. Materials and Methods

### 2.1. Collection and Extraction of *R. minima* and *S. italica*

Leaf samples of *R. minima* and *S. italica*, both one and a half years old, were collected from the University of Sharjah Garden, United Arab Emirates, during two distinct seasons based on recorded meteorological temperature data. The first collection was conducted in August 2021, during the summer season, when temperatures reached a maximum of 45°C, and relative humidity ranged between 79% and 94%. The second collection took place in early February 2022, during the winter season, when temperatures dropped to a minimum of 7°C, with humidity levels reaching 94%–100%. All plants were grown under natural light and environmental conditions in the same open garden setting to ensure uniform exposure to sunlight and ambient humidity. Although recorded using official data from the Sharjah Airport meteorological station, enabling us to interpret observed metabolic shifts in the context of naturally occurring environmental stressors. The site is representative of typical arid-zone habitats in the UAE, with sandy soils characterized by low moisture retention and limited organic content, based on direct field observation and prior documentation of the local edaphic conditions.

Healthy, mature leaves were carefully selected from multiple plants of each species to ensure a representative sample. The collected leaves were first cleaned with distilled water to remove any surface impurities and then air-dried at room temperature. Once dried, the leaves were finely powdered using a TissueLyser II (Model number 1218200424E, Qiagen, Hilden, Germany). Then, 10 g of the powdered leaf materials was soaked in 50 mL of absolute ethanol and shaken for 24 h at 90 rpm in a shaking incubator to ensure thorough extraction of bioactive compounds. Later, the mixture was sonicated in a water bath for 2 h at room temperature to enhance extraction efficiency. The resulting extract was filtered through a 0.45-μm syringe filter to remove solid residues. Finally, ethanol was completely evaporated under reduced vacuum and kept in the fridge for further analysis.

### 2.2. Total Phenolic Content (TPC) Assay

Phenolic contents were quantified using the Folin–Ciocalteu reagent [17]. Briefly, in 96-well plates, 10 μL of the plant extracts (3 mg/mL) was mixed with 100 μL of 10 × diluted Folin–Ciocalteu reagent, reaching final concentrations of 30 μg/mL. The resulting solutions were mixed and kept in the dark at room temperature for 5 min. After that, 90 μL of 7.5% saturated sodium carbonate solution was added and incubated at room temperature in the dark for another 2 h. The absorbance was measured at 750 nm using a microplate reader (Crocodile, Titertek Berthold, Bad Wildbad, Germany). A calibration curve was generated using gallic acid standards at concentrations of 3, 6, 12.5, 25, 50, 75, 100, 150, 300, and 400 μg/mL (Supporting Figure 2). The phenolic content of plant extracts was expressed in gallic acid equivalents (GAE) and analyzed using Excel and GraphPad Prism 5.0 (GraphPad Software, La Jolla, CA, USA.

### 2.3. Total Flavonoid Content (TFC) Assay

TFC in the extracts was determined using an aluminum chloride colorimetric assay [18]. To generate the standard calibration curve, quercetin standard solutions (20, 40, 60, 80, and 100 μg/mL) were prepared in absolute methanol (Supporting Figure 3). Then, 50 μL of plant extracts (1 mg/mL) was combined with 10 μL of 10% aluminum chloride solution and 150 μL of 96% ethanol for analysis. In a 96-well plate, 10 μL of 1 M sodium acetate was added to the mixture. A blank was prepared by including all the reagents and then mixed and incubated at room temperature for 40 min in a light-protected environment. A microplate reader (Versamax Microplate Reader, USA) was used to determine the absorbance at 415 nm. The calibration curve was used to calculate the TFC, which was expressed as mg of quercetin equivalent (QE)/g of *R. minima* and *S. italica* extracts. Triplicates of each extract and standard were used to determine the TFC.

### 2.4. GC–MS Analysis

Dried samples were treated with 20 μL of methoxyamine (20 mg/mL in pyridine) along with 50 μL of hexane. The mixture was vortexed and incubated in a water bath at 37°C for 1.5 h. Later, 90 μL of N-trimethylsilyl-N-methyl trifluoroacetamide (MSTFA) with 1% trimethylchlorosilane (TMS) was added and incubated at 37°C for 1 h [19, 20]. GC–MS analysis was performed using a GC-2010 gas chromatograph and a GCMSQP2010 Ultra GC-MS system equipped with an AOC-20i + 20s autosampler (Shimadzu, Tokyo, Japan) as described previously [21, 22]. The analysis was conducted using an Rtx5MS column (30 m length, 0.25 mm inner diameter, 0.25 μm film thickness; Restek, Bellefonte, Pennsylvania, USA), with 99.9% pure helium as the carrier gas at a column flow rate of 1 mL/min. The column temperature was programmed to start at 35°C for 2 min, followed by an increase of 10°C/min until reaching 250°C. Subsequently, the temperature was raised by 20°C/min until reaching 320°C, where it was held for 23 min. Samples were injected in splitless mode, with an injection volume of 1 μL and an injection temperature of 250°C. The mass spectrometer was operated in electron impact mode at 70 eV, with ion source and interface temperatures of 240°C and 250°C, respectively. The MS mode was set to scan at a speed of 1428 m/z over a mass range of 35–450 m/z. Data acquisition and processing were performed using Shimadzu's MSD Enhanced ChemStation software. Compound identification was achieved by comparing the measured fragmentation patterns with those in the National Institute of Standards and Technology (NIST) 14 Mass Spectral Library [23].

### 2.5. Antioxidant Assays

A ROS-based method was used to assess antioxidant activity following the approach described by Al-Nablsi et al. [24–26]. The absence of ROS and free radicals in a tested sample indicates the presence of an antioxidant. TPC, TFC, and 2,2-diphenyl-1-picryl-hydrazyl-hydrate (DPPH) free radical scavenging assays were used to determine the antioxidant activity of *R. minima* and *S. italica* extracts.

#### 2.5.1. DPPH Assay

Free radical scavenging activity of all leaf samples was evaluated using DPPH radical scavenging assay, following a previously adapted protocol [27]. The DPPH reagent was prepared in methanol at a concentration of 0.08 mg/mL, while methanolic extracts of the leaf samples and the positive control (butylated hydroxytoluene, BHT) were prepared at varying concentrations (1, 10, 50, 100, 250, and 500 μg/mL). Triplicates of each extract were added to a 96-well plate, where 50 μL of each extract was mixed with 50 μL of the DPPH reagent, with a negative control of DPPH in methanol. The plate was incubated in the dark for 60 min at room temperature. DPPH reagent changed color from dark violet to light yellow, indicating the reduction of free radicals by antioxidants in the extracts. Absorbance was measured at 515 nm using a microplate reader (Epoch 2 Microplate Spectrophotometer, BioTek Instruments, Inc., Winooski, VT, USA), and the resulting data were analyzed in Excel to calculate the percentage of free radical scavenging activity based on the decrease in DPPH absorbance.

The scavenging activity was calculated using the following equation:

(1) $$\begin{array}{c} \text{Scavenging }\%\text{ of DPPH}=\left(\frac{\text{Abs negative control}-\text{Abs sample}}{\text{Abs negative control }}*100\right). \end{array}$$

### 2.6. Metabolic Profiling and Meta-Analysis of *R. minima* and *S. italica*

Multivariate statistical analysis was performed using MetaboAnalyst 6.0 available at https://www.metaboanalyst.ca/, enrichment pathway analysis, principal component analysis (PCA), partial least squares-discriminant analysis (PLS-DA), and hierarchical clustering were performed to explore the patterns and pathways affected by the metabolites. The identified metabolites were first exported to an Excel datasheet for further filtering. The metabolite concentrations were represented using area percentage. The complete metabolic profiles of both species were screened to identify shared metabolites as well as metabolites unique to each plant. First, unique metabolites present in only one plant species but absent in the other were identified, along with metabolites showing increased concentration in the winter season. Based on their uniqueness, the metabolites specifically present at higher levels in the winter season were further analyzed. Additionally, metabolites unique to one season and absent in the other within the same plant species were identified. These unique and seasonally more abundant metabolites were further subjected to pathway analysis to explore their biological and functional significance. The chemical profiles of phenolic compounds and flavonoid content were analyzed to evaluate the metabolic variations between *R. minima* and *S. italica*.

### 2.7. Statistical Analysis

The data consisted of three biological replicates, each measured in triplicate (three technical replicates), and the average of the three technical replicates was used for statistical evaluation. Prior to analysis, data normality was assessed. Univariate analysis was conducted using GraphPad Prism (v5.04, La Jolla, CA, USA) to complement the multivariate findings. Two-way analysis of variance (ANOVA) was used to evaluate the statistical significance of metabolite differences between groups, *F*_1,10_, indicating 1 and 10 degrees of freedom for between- and within-group comparisons ensuring rigorous validation of the results. This comprehensive workflow provided insights into the metabolic alterations and potential biomarkers specific to the studied groups. Statistical significance was calculated with multiple comparisons test, and significance levels were indicated by asterisks (^∗^, *p* < 0.05; ^∗∗^, *p* < 0.01; ^∗∗∗^*p* < 0.001; ^∗∗∗∗^*p* < 0.0001).

## 3. Results

### 3.1. Metabolic Variation of *R. minima* and *S. italica* Is Seasonal and Species-Specific

**Figure 1:**
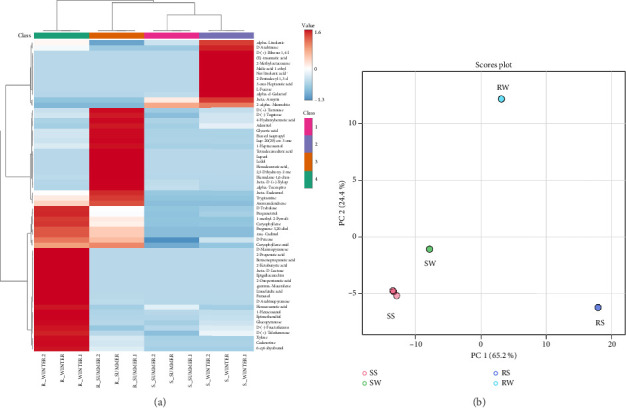
Metabolic profile analysis. (a) Heatmap analysis of the metabolic profiles in *R. minima* and *S. italica* in both seasons (winter and summer). Red color indicates highly expressed, and blue color indicates low expression. (b) Principal component analysis of *R. minima* and *S. italica* metabolite profiling in both seasons.

The metabolic analysis of *R. minima* and *S. italica* plant extracts revealed a distinct metabolic variation associated with seasonal changes (Supporting Figure 1). Metabolic heatmap of the top 50 metabolites showed a distinct clustering between summer and winter of both *R. minima* and *S. italica* (Figure 1(a)). *R. minima* displayed a higher abundance of metabolites clustering in winter, reflecting a lower temperature stress response, while summer shows lower levels. In *S. italica*, moderate metabolic changes occur, with increased levels of specific metabolites in winter and reduced chemical diversity in summer (Figure 1(a)). This pattern may reflect stronger metabolic adaptation of *R. minima* to winter stress compared to *S. italica*. PCA score plot shows clear separation between the samples based on seasonal and species-specific metabolic profiles. PC1 (65.2%) accounts for the majority of the variance, distinguishing *R. minima* (clustered on the right) from *S. italica* (clustered on the left). PC2 (24.4%) further separates the samples by season, with winter samples (Clusters 2 and 4) distinctly apart from summer samples (Clusters 1 and 3). This separation highlights significant metabolic differences driven by both species and seasonal factors (Figure 1(b)).

### 3.2. Comparative Analysis of the Phenolic Metabolites of Both Medicinal Plants *R. minima* and *S. italica*

**Figure 2:**
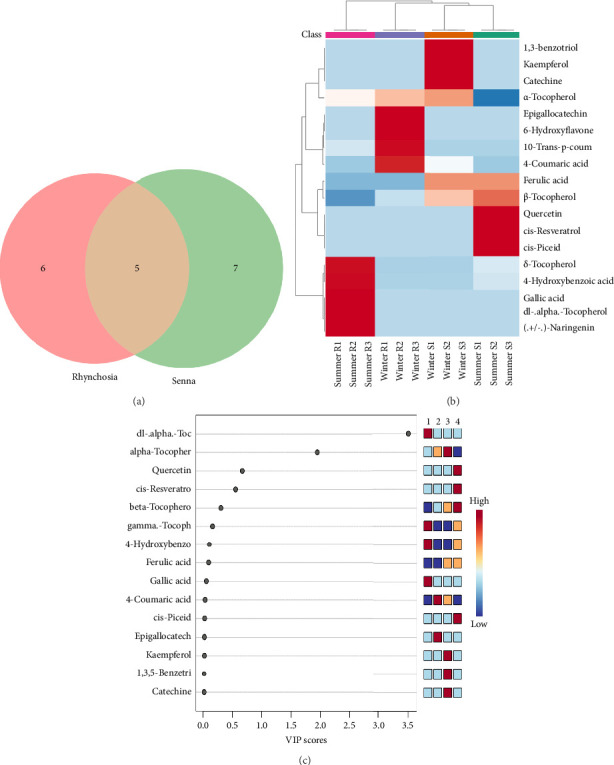
(a) Venn diagram of phenolic compounds in *R. minima* and *S. italica* showing the number of unique and shared metabolites. (b) Heatmap analysis of the phenolic compounds in *R. minima* and *S. italica* in winter and summer. Red color indicates highly expressed, and blue color indicates low expression. The values of phenolic contents represented the area under the peak in each group. Euclidean distance was chosen for similarity measure, and ward's linkage was used for clustering. (c) VIP score of phenolic compounds in *R. minima* and *S. italica,* with red and dark red intensities indicating compound abundance. The groups are categorized as: 1: summer *R. minima*, 2: winter *R. minima*, 3: summer *S. italica*, and 4: winter *S. italica.* VIP cutoff: VIP > 1 indicates key contributors, while VIP > 0.5 represents moderately important variables.

The phenolic profiles of *R. minima* and *S. italica* showcase both unique and shared metabolites, as illustrated by the Venn diagram (Figure 2(a)). *S. italica* has 7 unique compounds that were absent in *R. minima*, including cis-resveratrol, quercetin, cis-piceid, kaempferol, catechin, 1,3,5-benzetriol, and ferulic acid, while *R. minima* contains six unique phenolic compounds that were absent in *S. italica*, such as dl-α-tocopherol, (+/−)-naringenin, epigallocatechin, 6-hydroxyflavone-β-*D*-glucoside, 1-*O*-trans-*p*-Coumaroylglycerol, and gallic acid. Both plants share 5 common compounds: α-tocopherol, β-tocopherol, 4-coumaric acid, 4-hydroxybenzoic acid, and δ-tocopherol. The heatmap analysis of phenolic content highlighted distinct clustering and variations, reflecting seasonal and plant-specific differences. *R. minima* samples showed intense red coloration, indicating higher metabolite concentrations, suggesting an enriched bioactive profile compared to *S. italica* (Figure 2(b)). These metabolic variations reflect the unique ecological and functional adaptations of both species to environmental conditions, further reinforcing their potential medicinal applications.

### 3.3. VIP Score Analysis Reveals Key Phenolics in Sample Differentiation Related to Environmental Adaptation

VIP score of PLS-DA identifies dl-α-tocopherol and α-tocopherol had the highest scores (Figure 2(c)). Other key metabolites included quercetin, cis-resveratrol, β-tocopherol, and γ-tocopherol. These findings emphasize that tocopherols and flavonoids are important distinguishing metabolites in environmental adaptation of plants growing in harsh conditions.

### 3.4. Seasonal Variation Influences Phenolic Content, With Antioxidant Activity Peaking in Winter

Metabolic profile revealed a significant metabolic difference of the same plant in relation to seasons (Table 1). Analysis of phenolic content in *R. minima* shows notable seasonal variations. Notably, flavonoids 6-hydroxyflavone-β-D-glucoside and epigallocatechin were uniquely identified in *R. minima* growing in winter and completely absent in *S. italica* in both seasons. Similarly, 1-*O*-trans-*p*-coumaroylglycerol was exclusively detected in the winter *R. minima* plant. Additionally, 4-coumaric acid and β-tocopherol showed slightly increased levels in winter *R. minima* plant compared to summer. Additionally, α-tocopherol was dominant in winter *R. minima* and in summer *S. italica*. Summer *R. minima* plant contains unique compounds, such as gallic acid, (+/−)-naringenin, and dl-α-tocopherol, with no phenolic compounds significantly increasing compared to winter.

Meanwhile, during winter, *S. italica* is characterized by unique and elevated levels of quercetin with moderate VIP score, cis-resveratrol, and cis-piceid. β-Tocopherol, a shared compound, shows a notable increase in winter, while ferulic acid maintains consistent levels across both seasons. In contrast, for summer, *S. italica* with unique flavonoids such as kaempferol, catechin, and 1,3,5-benzetriol is prominent (Figure 2). Therefore, predicted activity suggests that winter conditions enhance the accumulation of bioactive phenolic compounds in *R. minima* and hence will exhibit a strong bioactivity, particularly in stress defense and antioxidant capacity. Similarly, winter *S. italica* benefits from its antioxidant and anti-inflammatory-rich profile, while summer *S. italica* may still retain antioxidant potential but with a distinct flavonoid composition. These findings highlight seasonal influences on phytochemical composition, with winter favoring a higher concentration of bioactive phenolic compounds.

### 3.5. Comparative Analysis of Flavonoid Contents

Flavonoid variation between plants and within the same plant species due to seasonal changes reveals significant adaptive responses. In *R. minima*, flavonoid content is relatively higher in winter than summer; however, it remains lower than the winter levels in *S. italica*. Flavonoid contents align with seasonal variation, with *S. italica* exhibiting the highest flavonoid concentration during winter, primarily due to the high abundance of unique flavonoid such as quercetin, a potent antioxidant flavonoid. This emphasizes the plant adaptive response to winter stress through the production of bioactive compounds.

**Figure 3:**
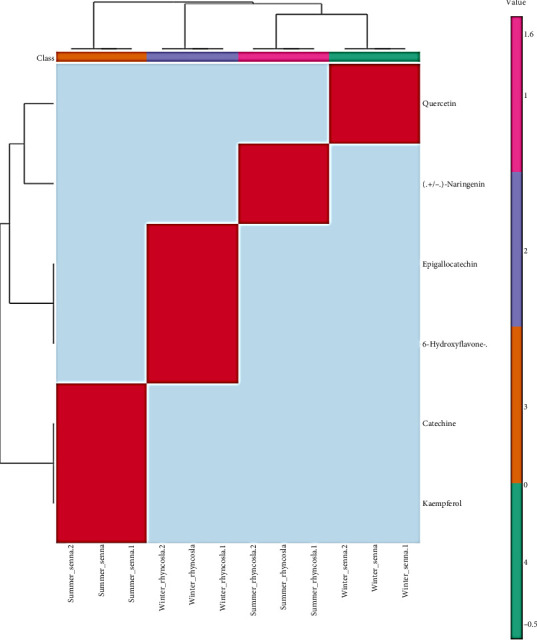
Heatmap analysis of flavonoid profiles in *S. italica* and *R. minima* in both seasons (winter and summer). Red color indicates highly expressed, and blue color indicates low expression, where the sample colors were orange: summer *S. italica*, purple: winter *R. minima*, pink: summer *R. minima*, and green: winter *S. italica*.

*R. minima* in winter shows a moderate increase in flavonoid content, driven by the accumulation of unique flavonoids as epigallocatechin and 6-hydroxyflavone-β-*D*-glucoside, which enhance its antioxidant defense. During summer, *R. minima* produce only (+/−)-naringenin, resulting in comparatively lower flavonoid levels. Meanwhile, *S. italica* continues to biosynthesize kaempferol and catechin during summer, but their combined concentrations are lower compared to the high levels of quercetin in winter (Figure 3). These findings highlight the role of specific flavonoids, particularly quercetin in *S. italica*, in shaping seasonal variations in flavonoid content and their ecological significance.

### 3.6. Comparative Analysis of Flavonoid and Phenolic Contents and Antioxidant Activity in Relation to Seasons

**Figure 4:**
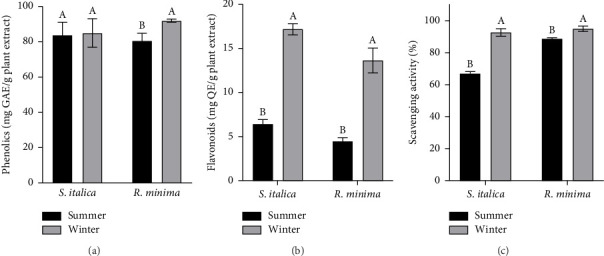
(a) Seasonal variation in total phenolic content of *R. minina* and *S. italica* ethanolic extracts (mean ± SD). (b) Seasonal variation in total flavonoid content of *R. minina* and *S. italica* ethanolic extracts (mean ± SD). (c) Seasonal variation in the scavenging activity for antioxidant activity of *R. minina* and *S. italica* ethanolic extracts (mean ± SD).

The TPC in *R. minina* was 4.8% higher in winter than in summer (fold change: 1.05, *F*_1,10_ = 21.237, *p* ≤ 0.001). In contrast, *S. italica* showed no significant seasonal variation, with phenolic content maintained between summer (78 mg GAE/g) and winter (80 mg GAE/g) (*F*_1,10_ = 0.082), respectively (Figure 4(a)). The season had a significant impact on the flavonoid content of both *R. minina* and *S. italica*, with *R. minima* displaying a strong effect (*F*_1,10_ = 39.271, *p* ≤ 0.001), while *S. italica* demonstrated an even more pronounced influence (*F*_1,10_ = 185.316, *p* ≤ 0.001). Flavonoid content was significantly higher in winter than in summer for both species. In *S. italica*, flavonoid content increased by 1.57-fold in winter compared to summer (from ∼7 mg/g to ∼18 mg/g), while in *R. minima*, it increased by 2.5-fold (from ∼4 mg/g to ∼14 mg/g). These results indicate that both species accumulate more flavonoids during winter, with *S. italica* showing a greater seasonal variation (Figure 4(b)).

The antioxidant activity of both *R. minina* and *S. italica* was significantly influenced by the season, with *R. minima* showing a moderate effect (*F*_1,10_ = 16.048, *p* ≤ 0.01) and *S. italica* exhibiting a stronger seasonal impact (*F*_1,10_ = 31.329, *p* ≤ 0.001). The scavenging activity, representing antioxidant activity, showed significant seasonal variation in both *R. minina* and *S. italica*. For *S. italica*, antioxidant activity increased from ∼75% in summer to 95% in winter, reflecting a 20% enhancement. In *R. minima*, antioxidant activity was consistently high, increasing from around 90% in summer to 97% in winter, a smaller but notable 7% increase. Such results suggest that while both species have high antioxidant activity during the winter, *S. italica* displays a more noticeable seasonal variation compared to the relatively consistent activity of *R. minima* (Figure 4(c)).

Antioxidant activity showed a significant seasonal variation in both *S. italica* and *R. minina*. For *S. italica*, IC_50_ decreased from 144.89 μg/mL in summer to 84.25 μg/mL in winter, reflecting a 41.8% improvement in antioxidant activity during winter (lower IC_50_ values indicate higher antioxidant activity). Similarly, for *R. minima*, IC_50_ decreased from 98.34 μg/mL in summer to 68.47 μg/mL in winter, indicating a 30.4% enhancement in antioxidant activity. These results demonstrate that both species exhibit stronger antioxidant activity in the winter, with *S. italica* showing a more pronounced seasonal change than *R. minima* (Table 2).

### 3.7. Enriched Pathways in *R. minima* and *S. italica* Winter Based on Unique Seasonable Winter Metabolites

**Figure 5:**
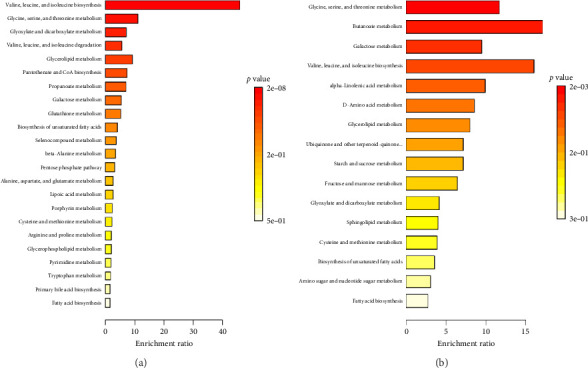
Metabolite set enrichment analysis (MSEA) ranked by holm *p* value using GC–MS/MS data of the winter samples showed the top enriched pathways for (a) unique winter metabolites in *R. minima* based on sorting out the GC–MS/MS data. (b) Unique winter metabolites in *S. italica* based on sorting out the GC–MS/MS data. Red color: low *p* value (high significance), yellow: high *p* value (low significance). Pathway enrichment analysis was conducted using MetaboAnalyst by uploading significant metabolites to the pathway analysis module with KEGG-based enrichment and topology analysis.

Both *R. minima* and *S. italica* plants exhibited an increase in enriched metabolic pathways during winter, particularly in central carbon metabolism and amino acid biosynthesis. Notably, they exhibit significant enrichment in valine, leucine, and isoleucine biosynthesis, which supplies essential branched-chain amino acids, which supports protein synthesis and stress adaptation. Glycine, serine, and threonine metabolism have been reported to enhance osmoprotection and antioxidant glutathione synthesis [28], while galactose metabolism supports carbohydrate mobilization for sustained energy [29]. Glyoxylate and dicarboxylate metabolism aid in carbon conservation, essential during reduced photosynthesis. Lipid-related pathways such as glycerolipid metabolism and the biosynthesis of unsaturated fatty acids ensure membrane stability and prevent oxidative damage [30, 31]. Additionally, *S. italica* exhibits strong antioxidative responses via ubiquinone and terpenoid-quinone biosynthesis, while *R. minima* relies more strongly on glutathione metabolism to neutralize ROS (Figure 5(a)). In *S. italica*, α-linolenic acid metabolism was also reported to be involved in oxylipin-mediated antioxidant regulation (Figure 5(b)). Overall, *R. minima* showed more diverse and intensified winter metabolic reprogramming, aligning with its higher antioxidant activity.

### 3.8. Enriched Pathways in *R. minima* and *S. italica* Summer Based on Unique Seasonable Summer Metabolites

**Figure 6:**
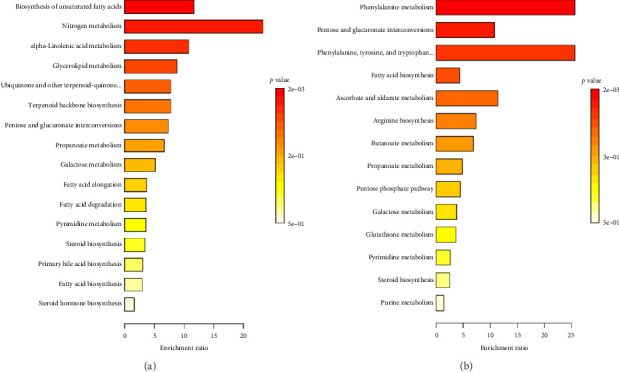
Metabolite set enrichment analysis (MSEA) ranked by holm *p* value showed the top enriched pathways. (a) Unique summer metabolites in *R. minima* based on sorting out the GC–MS/MS data. (b) Unique summer metabolites in *S. italica* based on sorting out the GC–MS/MS data. Red color: low *p* value (high significance), yellow: high *p* value (low significance). Pathway enrichment analysis was conducted using MetaboAnalyst by uploading significant metabolites to the pathway analysis module with KEGG-based enrichment and topology analysis.

The summer metabolic adaptations of *R. minima* and *S. italica* reveal shared and species-specific pathways for heat stress resilience, energy regulation, and oxidative stress management. Pentose and glucuronate interconversions and galactose metabolism are highly enriched in both species, emphasizing the role of carbohydrate metabolism in energy production and structural stabilization under high temperatures. Fatty acid biosynthesis is also prominent, ensuring membrane integrity and energy reserves. On the other hand, both plants showed unique antioxidative defenses, with *R. minima* utilizing ubiquinone and terpenoid-quinone biosynthesis, while *S. italica* relies on ascorbate and aldarate metabolism and glutathione metabolism for ROS detoxification. *R. minima* exhibit a strong emphasis on unsaturated fatty acid biosynthesis, α-linolenic acid metabolism, and fatty acid elongation and degradation, reflecting its strategy of lipid remodeling for membrane stability and oxidative stress defense. Additionally, nitrogen metabolism supports nitrogen storage and signaling, aiding in heat adaptation (Figure 6(a)). In contrast, *S. italica* shows a dominant enrichment in phenylalanine metabolism and phenylalanine, tyrosine, and tryptophan biosynthesis, highlighting its reliance on aromatic amino acid metabolism to produce secondary metabolites, such as flavonoids and alkaloids for UV protection and stress tolerance [32]. Propionate and butanoate metabolism further support lipid metabolism and energy balance in *S. italica* (Figure 6(b)). These results suggest that while both species activate protective mechanisms under summer stress, *R. minima* emphasizes lipid remodeling and energy pathways, whereas *S. italica* prioritizes biosynthesis of secondary metabolites.

## 4. Discussion

In the UAE, winter might introduce different environmental stresses, such as higher humidity, lower rainfall, changes in UV radiation, and susceptibility to herbivory attacks, all of which can influence plant metabolism and secondary metabolite production [33], and the colder conditions may enhance metabolic activity and flavonoid accumulation as plants shift from survival under extreme summer heat to optimizing growth and adaptation pathways [34]. This study highlights the seasonal metabolic adaptations of *R. minima* and *S. italica* to environmental stress, linking these changes to phenolic and flavonoid contents, which is valuable in metabolic analysis for pharmaceutical, functional food, and cosmetic applications due to its antioxidant and therapeutic properties [35]. PCA and heatmap analysis revealed clear species-specific metabolic variations, with *R. minima* showing a stronger winter shift and higher metabolite abundance, indicating greater cold stress adaptation. In contrast, *S. italica* exhibited moderate metabolic changes, revealing pronounced seasonal shifts in the plant's metabolic profile.

To further illustrate this, detailed identification of the phenolic metabolic contents reveals that *R. minima* and *S. italica* exhibit distinct seasonal phenolic adaptations, a finding further validated by biological activity analysis, which indicates that the phenolic content and potential efficacy of *R. minima* in winter are higher than those of *S. italica* during the same season. This difference may to be linked to the abundance of α-tocopherol, an important phenolic antioxidant that protects cell membranes by scavenging ROS, contributing to tolerance against cold stress [36–40]. While *S. italica* showed a decrease in α-tocopherol (vitamin E) during winter, *R. minima* showed an increase, suggesting that this seasonal boost in antioxidants gives *R. minima* an edge in medicinal potential and winter adaptability. Similarly, our finding aligns with studies, showing that higher α-tocopherol levels are associated with enhanced tolerance to winter stress, as observed in frost-tolerant wheat cultivars and other cold-adapted plants [37, 38, 41]. This increase was accompanied by a corresponding rise in antioxidant activity, indicating a seasonal adaptive response to environmental stressors [12, 42].

However, other species reinforce the notion that environmental cues drive the production of antioxidant metabolites in plants. For example, *Chelidonium majus* showed the highest TPC and free radical scavenging activity in the warmest part of the growing season [14], a trend analogous to the peak antioxidant levels observed in our study. Similarly, studies on maple trees have documented an evident seasonal variability in metabolite accumulation and antioxidant enzyme activities [43], supporting the general pattern that organisms elevate their antioxidant defenses in response to seasonal stress. It is worth noting that species-specific differences exist; in some plants, maximal antioxidant content occurs in early spring or at particular phenological stages (e.g., the rosette stage before flowering) rather than in midsummer [14]. Compared to these examples, our species exhibits its own unique timing for antioxidant phenolic peaks, suggesting that it has evolved a tailored seasonal response to its environment.

Additionally, flavonoid accumulation peaks in winter, with *S. italica* increasing 1.57-fold via quercetin for cold stress adaptation and *R. minima* rising 2.5-fold with epigallocatechin and 6-hydroxyflavone-β-D-glucoside. Both species reduce flavonoid biosynthesis in summer. The findings emphasize species-specific strategies for oxidative stress management and the significance of phenolic compound potency over quantity in seasonal adaptability. In *R. minima*, the presence of unique flavonoids such as 6-hydroxyflavone-β-D-glucoside and epigallocatechin is associated with scavenging free radicals and UV protection, critical for plant resilience in harsh winter conditions [44–46].

Similarly, *S. italica* exhibited markedly higher antioxidant activity in winter through the accumulation of unique compounds, such as cis-resveratrol, quercetin, and cis-piceid. Although *S. italica* did not show a statistically significant seasonal difference in TPC, it exhibited a markedly higher antioxidant activity in winter. This apparent discrepancy may be explained by the selective accumulation of potent nonphenolic antioxidants such as quercetin, cis-resveratrol, and tocopherols, which were uniquely detected in the winter metabolic profile. These compounds possess high radical scavenging capacity and can substantially enhance antioxidant activity independently of TPC [45, 47–49]. Additionally, synergistic interactions among antioxidant metabolites may amplify overall bioactivity [50], while even low concentrations of highly potent compounds can disproportionately contribute to antioxidant capacity [51]. Such compounds may also activate specific biochemical pathways or modulate stress responses, thereby strengthening the plant's adaptive defense mechanisms [52]. These findings underscore that antioxidant activity reflects not only compound quantity but also intrinsic potency and synergistic behavior—hallmarks of seasonal metabolic adaptation (Supporting Table 1).

Such variations suggest that plants bolster their antioxidant defenses when exposed to extreme conditions, likely to mitigate oxidative damage caused by high temperatures and intense solar radiation [53]. In other words, during harsh seasons, the plant's metabolism reallocates resources toward producing protective antioxidant compounds, which help counteract stress-induced ROS [54]. This dynamic metabolic adaptation aligns with previous findings that secondary metabolite production is influenced by seasonal fluctuations, optimizing stress tolerance and physiological homeostasis [55, 56].

Ecologically, the plant's ability to upregulate antioxidant metabolites during stressful seasons likely enhances its survival and fitness. By maintaining oxidative balance when conditions are most extreme [12], the plant can prevent cellular damage and ensure critical physiological processes (such as growth or reproduction) are not compromised, illustrating the advantage of a flexible metabolism in adaptation to environmental stress. This pattern has been observed in various species, where secondary metabolite levels (such as phenolics and flavonoids) are increased during environmentally challenging periods, boosting the plant's defense mechanisms [57, 58]. From a medicinal standpoint, the seasonal surge in bioactive compounds means that this species could be a particularly rich source of natural antioxidants at certain times of the year. For instance, if phenolic and flavonoid contents peak in winter, harvesting the plant during that period could yield extracts with maximized antioxidant potency [14, 59]. Studies indicate that seasonal fluctuations significantly impact the nutritional and therapeutic properties of medicinal plants, affecting their antioxidant and anti-inflammatory efficacy [60]. Such insights are valuable for both conservation and utilization: timing the collection of plant material to coincide with high bioactivity can improve the efficacy of herbal preparations or nutraceutical products [61].

Moreover, the varieties of metabolites that aid the plant's stress adaptation have well-known health benefits in humans, including antioxidative, antimicrobial, and anticancer effects [15, 62]. Multiomics, particularly metabolomics, is vital for optimizing crop breeding, harvest timing, and bioactive compound production. It enables the identification of seasonal metabolic shifts, ensuring the extraction of bioactive compounds at their peak potency [63]. By mapping plant-specific metabolic adaptations, metabolomics enhances targeted breeding strategies for improved phytochemical profiles [15, 62]. Additionally, the creation of metabolome databases facilitates precision agriculture, aiding in crop conservation and sustainable utilization [64].

Enrichment analysis identified key metabolic pathways that drive the observed seasonal adaptations in both *R. minima* and *S. italica*, reinforcing the correlation between metabolic shifts and antioxidant activity. The analysis highlighted that *R. minima* exhibits a pronounced seasonal variation with a broader range of enriched pathways across both seasons, particularly in amino acid metabolism, which plays a crucial role in stress adaptation and is enriched during colder conditions. Additionally, the enrichment in antioxidant-related pathways, such as glutathione metabolism, is aligned with the observed increase in antioxidant activity. This confirms that seasonal shifts are not merely a result of variations in individual compounds but are driven by overarching metabolic adjustments [65, 66]. This was mirrored by the increased levels of α-tocopherol and β-tocopherol in *R. minima*. The summer showed improved lipid biosynthesis, as saturated and unsaturated fatty acids highlight lipids' role in remodeling to combat heat stress. Antioxidant pathways, such as ubiquinone and terpenoid-quinone biosynthesis, reduce the resulting oxidative damage.

Conversely, *S. italica* primarily enhances α-linolenic acid metabolism and contributes to oxylipin signaling, which regulates the expression of antioxidant enzymes [67, 68]. In addition, enriched ubiquinone and other terpenoid metabolism align with the higher TFC such as quercetin and antioxidant activity observed during winter [69], thereby optimizing oxidative stress protection and ensuring membrane fluidity under cold stress [70]. However, in summer enriched pathway, ascorbate–glutathione metabolism has been shown to scavenge ROS and enhance stress tolerance, which is particularly crucial for *S. italica* in extreme conditions [71]. These findings suggest a comprehensive strategy for maintaining homeostasis under seasonal environmental stresses, highlighting the interplay between metabolism, antioxidant defense mechanisms, and energy regulation, which were confirmed through metabolic profiling and experimental assays to correlate directly with the observed antioxidant activity [72, 73]. This seasonal enrichment not only supports the observed differences in flavonoid content and antioxidant activity but also underscores the species-specific metabolic strategies that these plants employ to maintain homeostasis in response to fluctuating environmental conditions [74].

While this study uncovered clear seasonal patterns in metabolite levels and antioxidant activity, it was limited to a specific timeframe and set of conditions. We did not capture potential year-to-year variations or the influence of different microhabitats, so the generality of these patterns across varying environmental contexts remains uncertain. Future studies should incorporate multiyear data and diverse locations to confirm the consistency of the observed seasonal trends. Another limitation is that our analysis focused on broad classes of compounds (total phenolics, total flavonoids, etc.) rather than identifying individual metabolites. Notably, we observed no strict one-to-one correlation between the TPC or TFC and the antioxidant capacity of the extracts [14]. This implies that particular compounds (or synergistic combinations of compounds) drive the antioxidant effects. Isolating and characterizing new specific bioactive molecules will be an essential next step to pinpoint which components are most responsible for the plant's antioxidant activity. Furthermore, this study did not explore the molecular mechanisms that regulate these seasonal metabolic changes. Investigating the genetic and enzymatic pathways that underline the observed variations would provide insight into how the plant senses environmental cues and modulates its metabolism accordingly. Addressing these questions in future research will build upon our results and helpfully elucidate how this species balances ecological survival with the production of medicinally beneficial compounds through seasonal metabolic adaptation.

## 5. Conclusion

This study underscores the critical role of seasonal metabolic variation in *R. minima* and *S. italica*, highlighting how these medicinal plants adjust their antioxidant defense systems to withstand the external environmental stresses of temperature and humidity. Metabolic profiling and antioxidant assays revealed clear seasonal shifts in the accumulation of bioactive secondary metabolites, such as phenolics and flavonoids, in the winter, which are essential for protecting plant cells from oxidative damage. Enrichment analysis indicated that these changes are driven by specific metabolic pathways, with each species employing distinct survival strategies in response to winter stress. *R. minima* exhibited a stronger metabolic adaptation, characterized by glutathione metabolism, amino acid biosynthesis, and enhanced redox buffering, while *S. italica* showed seasonal peaks in quercetin, and *R. minima* accumulated α-tocopherol, suggesting targeted harvesting strategies to maximize their medicinal value for nutraceutical and pharmaceutical applications. These differences highlight the species-specific biochemical strategies that underpin resilience in arid environments. This study enhances our understanding of plant resilience and metabolic flexibility, paving the way for advances in agriculture, conservation, and medicine. Future research should investigate the molecular mechanisms underlying these adaptations and explore the potential health benefits of the identified bioactive compounds.

## Figures and Tables

**Table 1:** Phenolic analysis in *R. minima* and *S. italica* at different seasons.

Phenolic compound	*R. minima*		*S. italica*	
	Winter	Summer	Winter	Summer
α-Tocopherol	+++	+	+++	++
β-Tocopherol	+	+	++	+
4-Coumaric acid	+	−	−	+
6-Hydroxyflavone-β-D-glucoside	+	−	−	−
Epigallocatechin	+	−	−	−
cis-Resveratrol	−	−	+	−
Quercetin	−	−	++	−
4-Hydroxybenzoic acid	−	+	+	−
cis-Piceid	−	−	+	−
Kaempferol	−	−	−	+
Catechin	−	−	−	+
1,3,5-Benzetriol	−	−	−	+
dl-α-Tocopherol	−	+++	−	−
(+/−)-Naringenin	−	+	−	−
Gallic acid	−	+	−	−
Ferulic acid	−	−	+	+
1-*O*-trans-*p*-Coumaroylglycerol	++	+	−	−
γ-Tocopherol	−	++	+	−

*Note:* “+++” = highest concentration, “++” = increased presence, “+” = present, “−” = not detected.

**Table 2:** Seasonal variation of antioxidant activity of *R. minima* and *S. italica* ethanolic extracts (calculated by in IC_50_ values in μg/mL of the mean ± SD).

Plant species	Summer	Winter
*S. italica*	144.89 ± 5.23	84.25 ± 4.80
*R. minima*	98.34 ± 8.55	68.47 ± 7.984

## Data Availability

The data that support the findings of this study are available from the corresponding author upon reasonable request.

## Supporting Information

Supporting Figure 1 presents representative GC–MS chromatograms of the phytochemical profiles of *R*. *minima* and *S*. *italica*. Supporting Figures 2 and 3 show the standard calibration curves for gallic acid and quercetin, respectively. Supporting Table 1 summarizes key winter phenolic metabolites in both species, highlighting their antioxidant roles.
