# Supplementary material for: Seasonal Metabolic Adaptations and Antioxidant Defense Mechanisms in the Resilience of Rhynchosia minima and Senna italica Legumes in Arid Region
Source: Scientifica (Cairo). 2025 Sep 10;2025:1359373. doi: 10.1155/sci5/1359373 (PMC12443509; doi:10.1155/sci5/1359373)
Supplement: Supporting Information — Additional supporting information can be found online in the Supporting Information section. [file 1359373.f1.docx]

**Supplementary Figure 1**. Representative GC-MS chromatograms showing seasonal variation in the phytochemical profiles of *Rhynchosia minima* and *Senna italica.*Black: *R. minima* (Summer), Pink: *R. minima* (Winter), Blue: *S. italica* (Summer), Red: *S. italica* (Winter).

**Supplementary Figure 2**. The standard calibration curve for gallic acid was linear within the tested range, with the equation *y =* .001284*X + 0.02309, and a correlation coefficient (R²) of 0.9781.

**Supplementary Figure 3**. The standard calibration curve for quercetin was linear within the tested range, with the equation *y* = 0.008321*X + 0.06443 and a correlation and a coefficient (R²) of 0.9944.

**Supplementary Table 1.** Key winter phenolic metabolites and their antioxidant roles.

| **Compound** | **Increased in Plant** | **Antioxidant Role** | **Enriched Pathway in Plant** | **Ref** |
| --- | --- | --- | --- | --- |
| α-Tocopherol | *R. minima* (Winter) | A potent lipophilic antioxidant that protects cell membranes by scavenging ROS. Increased amount associated with tolerance to winter stress. | Tocopherol biosynthesis | (El Kayal et al. 2006; Janeczko et al. 2018; Müller et al. 2006) |
| β-Tocopherol | *R. minima* (Winter) | It scavenges lipid peroxyl radicals to maintain membrane integrity. | Tocopherol biosynthesis | (Azzi 2004; El Kayal et al. 2006; Janeczko et al. 2018; Mateos et al. 2003; Štolfa Čamagajevac et al. 2018) |
| 4-Coumaric acid | *R. minima* (Winter) | A phenolic acid with antioxidant and anti-inflammatory activity, protects against oxidative stress. | Phenylpropanoid pathway | (Al-Nablsi et al. 2022; Guglielmi et al. 2003; Solecka et al. 1999) |
| 6-Hydroxy flavone-β-D-glucoside | *R. minima* (Winter) | Flavonoid pathway, providing antioxidant and UV protection. | Flavonoid biosynthesis | (Chaves and Escudero 1999; Li et al. 2023) |
| Epigallocatechin | *R. minima* (Winter) | A flavonoid known for strong antioxidant effects; scavenges free radicals effectively. Adaption role in cold stress in plant and environmental interaction. | Flavonoid biosynthesis | (Ahammed et al. 2023; Li et al. 2023; Wang et al. 2022) |
| cis-Resveratrol | *S. italica* (Winter) | It protects against oxidative stress and inflammation. | Stilbene biosynthesis | (Dubrovina and Kiselev 2017; Truong et al. 2018) |
| Quercetin | *S. italica* (Winter) | Flavonoid with strong antioxidant increased as response of low temperature, reduces oxidative damage, inflammation and enhance cold resistance in plant. | Flavonoid biosynthesis | (Agati et al. 2012; Li et al. 2023; Liu et al. 2025; Zhao et al. 2024) |
| 4-Hydroxybenzoic acid | *S. italica* (Winter) | Antioxidant and anti-inflammatory; prevents oxidative damage in tissues and mitigate freezing stress. | Phenolic acid pathway | (Horváth et al. 2007; Zhang et al. 2012) |
| cis-Piceid | *S. italica* (Winter) | Resveratrol derivative with similar antioxidant properties; prevents oxidative stress. | Stilbene biosynthesis | (Balestrazzi et al. 2011; Dubrovina and Kiselev 2017; Valletta et al. 2021) |
| Ferulic acid | Both seasons | Antioxidant: scavenges free radicals and protects against UV-induced oxidative stress. | Phenolic acid pathway | (Król et al. 2015; Pennycooke et al. 2005; Zduńska et al. 2018) |
